# Statistical Regression Model of Water, Sanitation, and Hygiene; Treatment Coverage; and Environmental Influences on School-Level Soil-Transmitted Helminths and Schistosome Prevalence in Kenya: Secondary Analysis of the National Deworming Program Data

**DOI:** 10.4269/ajtmh.20-1189

**Published:** 2021-04-12

**Authors:** Collins Okoyo, Suzy J. Campbell, Chrispin Owaga, Nelson Onyango, Graham Medley, Charles Mwandawiro

**Affiliations:** 1 Eastern and Southern Africa Centre of International Parasite Control, Kenya Medical Research Institute (KEMRI), Nairobi, Kenya;; 2 School of Mathematics, College of Biological and Physical Sciences, University of Nairobi, Nairobi, Kenya;; 3 Deworm the World, Evidence Action, Washington, District of Columbia;; 4 Deworm the World, Evidence Action, Nairobi, Kenya;; 5 Faculty of Public Health and Policy, London School of Hygiene and Tropical Medicine, London, United Kingdom

## Abstract

According to the Kenya National School-Based Deworming program launched in 2012 and implemented for the first 5 years (2012–2017), the prevalence of soil-transmitted helminths (STH) and schistosomiasis substantially reduced over the mentioned period among the surveyed schools. However, this reduction is heterogeneous. In this study, we aimed to determine the factors associated with the 5-year school-level infection prevalence and relative reduction (RR) in prevalence in Kenya following the implementation of the program. Multiple variables related to treatment, water, sanitation, and hygiene (WASH) and environmental factors were assembled and included in mixed-effects linear regression models to identify key determinants of the school location STH and schistosomiasis prevalence and RR. Reduced prevalence of *Ascaris lumbricoides* was associated with low (< 1%) baseline prevalence, seven rounds of treatment, high (50–75%) self-reported coverage of household handwashing facility equipped with water and soap, high (20–25°C) land surface temperature, and community population density of 5–10 people per 100 m^2^. Reduced hookworm prevalence was associated with low (< 1%) baseline prevalence and the presence of a school feeding program. Reduced *Trichuris trichiura* prevalence was associated with low (< 1%) baseline prevalence. Reduced *Schistosoma mansoni* prevalence was associated with low (< 1%) baseline prevalence, three treatment rounds, and high (> 75%) reported coverage of a household improved water source. Reduced *Schistosoma haematobium* was associated with high aridity index. Analysis indicated that a combination of factors, including the number of treatment rounds, multiple related program interventions, community- and school-level WASH, and several environmental factors had a major influence on the school-level infection transmission and reduction.

## INTRODUCTION

Soil-transmitted helminths (STH), including *Ascaris lumbricoides*, *Trichuris trichiura*, and the hookworms (*Necator americanus* and *Ancylostoma duodenale*), and also schistosomes (*Schistosoma mansoni* and *Schistosoma haematobium*) are among the neglected tropical diseases (NTDs) earmarked for global elimination by 2030 by the World Health Organization (WHO).^1^ According to global estimates, STH and schistosomiasis are endemic in 166 and 76 countries, respectively.^2,3^ Combined, these infections affect more than three billion people globally, most of whom live in sub-Saharan Africa.^4^ Most of the endemic countries, including Kenya, have been implementing mass drug administration (MDA) programs using either school-based or community-based programs.^5–7^ Currently, preventive chemotherapy measures consist of annual or biannual mass treatments using albendazole or mebendazole for STH and praziquantel for schistosomiasis, based on assessed risk within each country.^8^

Kenya has been conducting a National School-Based Deworming (NSBD) program since 2012 by delivering treatment, through an MDA program, for STH and schistosomiasis to all school-aged children in all primary schools in 28 endemic counties spread across various regions of Kenya.^9–11^ The program’s impact on parasitological outcomes among the treated children has been closely monitored through a rigorous monitoring and evaluation (M&E) program, with the first 5 years of monitoring performed between 2012 and 2017.^8,9,12^ However, the impact of such a national large-scale program is usually known to be influenced by a variety of different factors, some of which are beyond the control of the program.^6^ Past modeling studies on STH and schistosome infection transmission dynamics have indicated that prevalence reduction as a direct consequence of treatment is influenced by the underlying intensity of the infection transmission (usually determined by the basic reproduction number, $R_{o}$), efficacy of the drugs used, and proportion of the overall population treated (i.e., treatment coverage).^6,13,14^ Furthermore, water, sanitation, and hygiene (WASH) availability as well as associated practices and behaviors have been shown to influence the rate of exposure to infectious materials in the environment (ova and larvae).^15^ In addition, the survival of free-living stages in the environment is influenced by various climatic and environmental factors.^16^

In this study, we assessed the factors associated with the school-level prevalence of STH and schistosome infections among the schools participating in the M&E program, a deviation from the commonly studied individual-level factors. Specifically, we aimed to investigate the effect of treatment, including the number of treatment rounds and coverage, community- and school-level WASH conditions, and environmental conditions around the school location. This is the first kind of investigation involving the inclusion of environmental factors in a large-scale national program within the country.

## MATERIALS AND METHODS

### Kenya National School-Based Deworming program context.

Kenya has been implementing a countrywide NSBD program since 2012, and is currently ongoing. The program aims to deworm, annually, all schoolchildren living in STH- and schistosomiasis-endemic subcounties to achieve elimination of these infections as a public health problem. The impact evaluation of the program is conducted independently by the Kenya Medical Research Institute (KEMRI) through a robust M&E program. The first 5 years (phase one) of the M&E program was implemented between 2012 and 2017,^8,9,12^ and the second phase is currently ongoing.^17^ The M&E program conducts impact evaluation through a series of repeat cross-sectional surveys in a representative, stratified, two-stage sample of schoolchildren across counties in Kenya to determine the national infection prevalence levels, as described in their first three surveys of phase one of the program: year 1, year 3, and year 5,^8,9,12^ and year 6 for phase two.^17^ During year 1 to year 5 surveys, an average of 199 schools per survey round in 16 counties in four regions: Western, Nyanza, Rift Valley, and Coast, were surveyed before treatment to measure the national infection levels. However, only 100 schools (five schools per county) were sampled during year 6 survey in 20 counties in six regions; Western, Nyanza, Rift Valley, Coast, Eastern, and North Eastern. In each of the sampled schools, 18 children (nine girls and nine boys) were sampled randomly from each of the six classes, including one early childhood development (ECD) class and classes 2–6, using random numbers, for a total of approximately 108 children per school. At each survey point, the program processed and examined in duplicate single stool or urine samples from each selected child for the identification of STH and schistosomiasis eggs using the Kato–Katz thick smear or urine filtration techniques.^8,18^ However, a comprehensive WASH questionnaire was only administered to all survey participants during the year 6 survey, with only limited WASH questions asked during year 5 survey.

### Current analysis framework.

The long-term observable impact of the NSBD program between year 1 and follow-up assessments leading to year 5 can be interpreted in terms of the following processes: 1) immediate infection reductions following yearly treatments: this depends on the drug efficacy and treatment coverage; 2) the rate of reinfection between the treatments, as explained by Nikolay et al.^6^; 3) the availability of the improved WASH conditions at school and home environments, as explained elsewhere^19^; 4) the environmental conditions that potentially influence the survival of free-living infectious materials in the environment; and 5) the availability of other complementary treatments by partner programs that deliver anthelminthic drugs. This analysis is based on a mixed-effects linear regression framework that incorporated key factors believed to be associated with a long-term infection impact. We therefore identified and assembled relevant indicator school-level data for all the sampled 199 schools in Kenya (Table 1).

**Table 1:** School and community WASH and environmental condition indicator components included in the analysis of factors associated with school-level soil-transmitted helminths and schistosome infection prevalence in Kenya

Indicator	Component	Source
Infection prevalence	Baseline (year 1) infection prevalence (%)	Baseline survey^9^
	Year 5 infection prevalence (%)	Year 5 survey^8^
	Prevalence relative reduction (%)	Year 5 survey^8^
Treatment data	Year 4 treatment coverage (%), summarized at the subcounty level	Evidence action
	Number of treatment rounds with albendazole since year 1	Evidence action and partner programs
	Number of treatment rounds with praziquantel since year 1	Evidence action and partner programs
	Classification of the areas according to whether they were treated by National School Based Deworming or partner programs	Evidence action and partner programs
Community-level WASH conditions	Household access to improved water source	Year 6 survey^17^
	Household access to any sanitation	Year 6 survey^17^
	Household access to handwashing facility with soap and water	Year 6 survey^17^
School-level WASH conditions	Type of water source (i.e., improved vs. unimproved)	Year 5 and 6 surveys^8,17^
	Type of sanitation (i.e., improved vs. unimproved)	Year 5 and 6 surveys^8,17^
	Availability of handwashing facility equipped with soap and water	Year 5 and 6 surveys^8,17^
	School population	Year 5 and 6 surveys^8,17^
	Pupil per latrine ratio (overall)	Year 5 and 6 surveys^8,17^
	Availability of other programs (e.g., school feeding program and sanitation program)	Year 5 survey^8^
	Latrine cleanliness and its structural integrity	Year 6 survey^17^
Environmental conditions	Land surface temperature (°C) (1 km resolution)	WorldClim^23^
	Aridity index (1 km resolution)	CGIAR Consortium for Spatial Information Figshare open data repository^28^
	Enhanced vegetation index (1 km resolution)	Moderate Resolution Imaging Spectroradiometer vegetation indices^26^
	Population density (population per 100 m^2^), (average projected population 2019) (100 m resolution)	WorldPop^29^
	Mean monthly precipitation (1 km resolution)	WorldClim^23^
	Elevation (30 m resolution)	RCMRD GeoPortal^24^
	Slope (30 m resolution)	Calculated from the elevation values
	Land cover (1 km resolution)	RCMRD GeoPortal^25^
	Soil type (250 m resolution)	International Soil Reference Information Centre world soil information^27^

RCMRD = Regional Center for Mapping of Resources for Development; WASH = water, sanitation, and hygiene.

### Data and data sources.

#### School-level infection prevalence.

Infection prevalence information for each school is usually recorded within the NSBD program by KEMRI during each survey round. The school’s infection prevalence was defined as the averaged infection prevalence observed among schoolchildren surveyed at that school (sample size of 108 children). In this analysis, we used two variables for school-level prevalence: prevalence data collected during baseline (year 1) survey, and that collected during the year 5 pretreatment survey, so as to enable the calculation of the prevalence relative reduction (PRR) over the 5-year period in the modeling. For each school, the PRR was derived as the difference in prevalence between year 1 and year 5 infection prevalence.

#### School-level treatment coverage.

Treatment coverage information for each school is usually recorded within the NSBD program, after each deworming activity, by Evidence Action: an international nongovernmental organization that provides technical support to the government’s NSBD program. School-level treatment coverage for each infection was aggregated and presented at the subcounty level. Treatment coverage was determined as the number of children who received treatment for each infection divided by the number targeted at each participating school. In addition, data on the number of treatment rounds since year 1 (2012) were accessed and compiled for each subcounty within the NSBD program geographic area. This required seeking permission to access data from other programs that distribute albendazole and praziquantel within the NSBD program geographic area; these programs included the Kenya lymphatic filariasis (LF) elimination program,^20^ *Tuangamize Minyoo Kenya Imarisha Afya* (TUMIKIA) project,^7^ and Schistosomiasis Consortium for Operational Research and Evaluation (SCORE) project.^21^ As a result, two variables were created, one which was a discrete variable recording the number of treatment rounds at the subcounty level within the whole NSBD geographic area, ranging from zero to seven rounds, and the other which categorized the treatment areas into two (i.e., areas treated by the NSBD program only, and areas treated by both NSBD and other partners).

#### School-level water, sanitation, and hygiene conditions.

Adequate access to appropriate WASH facilities may limit exposure of children to the infectious materials in the school environment.^15^ In this analysis, improved water source was defined as the proportion of the respondents with piped water into dwelling, piped water into yard/plot, public tap, boreholes, protected wells or springs, rain water collection, and bottled water, whereas improved latrine was defined as the proportion of the respondents with flush toilet, toilet connected to a piped sewer system, toilet connected to a septic system, flush to a pit latrine, pit latrine with slab, ventilated improved pit latrine, and composting toilet. These definitions are in accordance with those of the WHO/UNICEF Joint Monitoring Programme (JMP) for Water Supply, Sanitation, and Hygiene.^22^ The following school-level WASH factors were assessed: type of water source (improved versus unimproved), type of latrine (improved versus unimproved), availability of handwashing facility equipped with soap and water for washing of hands, school population, pupil per latrine ratio (overall and disaggregated by gender), latrine cleanliness and its structural integrity, and availability of other school programs such as school feeding and sanitation programs. Latrine cleanliness was assessed by the absence of strong smell, absence of visible feces on the latrine floor, and clean floor, whereas latrine structural integrity was assessed by the evidence of all the following: roof and walls with no holes, a functional lockable door, and a stable floor slab.^17^ Information on these school-level WASH conditions were collected during year 5 and 6 surveys by interviewing the pupils, head teachers, or visual inspection (Table 1). However, for some schools, some WASH conditions were missing, such as the availability of a handwashing facility (121 schools), and latrine cleanliness and its structural integrity (121 schools).

#### Household-level water, sanitation, and hygiene conditions.

In addition, adequate access to WASH conditions at the household level may limit the rate of reinfection after treatment; limited access to appropriate WASH conditions including sanitation at the household-level increases exposure of the children to the infectious materials.^19^ In this analysis, we included the following household-level WASH conditions as reported by the pupils during the year 6 survey: household access to improved water source, any type of household sanitation, and hygiene facility (i.e., handwashing facility equipped with soap and water) (Table 1). These variables were categorized as proportions of the children who reported access to these WASH conditions at their household. However, all these household-level WASH conditions were missing for 121 schools.

#### School-level environmental data.

Several environmental conditions have been determined as the potential influencers of the survival of free-living infectious materials in the environment, and hence they boost the transmission success of STH and schistosomiasis.^6,16^ In this analysis, we considered and extracted the following environmental factors, known to be influencers of helminth survival, from high-resolution satellite data from variety of sources and matched them to school locations: estimates of monthly average land surface temperature (LST) and precipitation at 30-arcsec (∼1 km) were downloaded from the WorldClim,^23^ elevation at 30 m resolution and land cover at 1 km resolution were obtained from the Regional Center for Mapping of Resources for Development (RCMRD) Geoportal,^24,25^ slope values were estimated from the elevation values, enhanced vegetation index (which measure vegetation density) at 1-km resolution was obtained from the Moderate Resolution Imaging Spectroradiometer,^26^ soil type at 250 m resolution was obtained from International Soil Reference Information Centre-World Soil Information,^27^ aridity index (AI) at 1 km resolution was obtained from the Consortium for Spatial Information (CGIAR-CSI),^28^ and population density (population per 100 m^2^) at 100 m resolution was obtained from WorldPop.^29^ These factors were documented and estimated for each school location by creating a buffer of 1 km around each school, with data averaged over the array of estimates (Table 1).

### Statistical analysis.

Regression analysis to determine the association with the impact of the deworming program was conducted in relation to several factors hypothesized to influence school-level infection prevalence in Kenya. The factors included in the analysis were variables related to treatment coverage and rounds, reported household-level WASH conditions, reported and observed school-level WASH conditions, and environmental conditions. A detailed description of all the variables included in this analysis is outlined in Table 1. Some schools had missing data for some of the covariates (as seen in the *Data and Data Sources* subsection); because those missing data could not be retrospectively collected, those schools were excluded from the analysis for those particular covariates only, and those covariates were investigated to determine if they had enough observations to warrant their inclusion in the respective models. Furthermore, all covariates were investigated to determine if they had sufficient observations/cell sizes needed to perform the analyses, and where insufficient observations were observed, the covariates were dropped.

Because we were interested in the factors directly associated with the school-level changes in the infection prevalence and PRR for both STH and schistosome infections, the outcome variables were defined at the school level as the year 5 averaged infection prevalence and PRR for each STH and schistosome species. The analysis was performed separately for each of these two outcomes of interest. School-level prevalence was defined as the average number of pupils infected with a particular infection over the total number examined (i.e., $\mathrm{School}\;\mathrm{prevalence}=\left[\frac{\mathrm{Number}\;\mathrm{of}\;\mathrm{pupils}\;\mathrm{positive}}{\mathrm{Number}\;\mathrm{of}\;\mathrm{pupils}\;\mathrm{examined}}\right]\times 100\%$) at that time point, and school-level PRR was defined as the difference in infection prevalence between year 1 and year 5 surveys (i.e., $\mathrm{PRR}=\left[\frac{\mathrm{Year}\;1\;\mathrm{prevalence}-\mathrm{Year}\;5\;\mathrm{prevalence}}{\mathrm{Year}\;1\;\mathrm{prevalence}}\right]\times 100\%$), where negative values of PRR indicated an increase in prevalence as opposed to RR. The association of the outcome variables with the outlined independent covariates was then modeled separately in a two-step approach: univariable and multivariable analyses using multilevel mixed-effects linear regression models with a random intercept at county and subcounty levels. A subcounty random intercept was included in the models because a number of schools were clustered within a given subcounty.

Factors associated with the school-level prevalence or PRR for each infection were first analyzed using univariable analysis, and covariates were considered for further analysis using multivariable mixed-effects linear regression if the 95% CIs of the coefficients did not include zero. To avoid collinearity in the multivariable models, the covariance of the selected variables was investigated pairwise to determine if any strong correlation (*r* ≥ 70 or *r* ≤ −0.70) existed among the variables. Three pairs of variables showed strong collinearity (AI was correlated with improved household water source, elevation was correlated with improved household water source, and elevation was correlated with LST) (Supplemental Table S1), and to ensure that no correlated pairs were included in the same model, we retained only covariates with the highest number of observations. We then developed the multivariable mixed-effects linear regression model for each infection using a sequential block-wise approach, where the variables found to be significant in the univariable analysis were included and eliminated one at a time until the most parsimonious model was obtained. The final model included only covariates whose 95% CIs of the coefficients did not include zero. For both univariable and multivariable models, negative values of the coefficients (*C*) indicated a decrease in the school-level infection prevalence or lower PRR, whereas positive values of the coefficients indicated an increase in the school-level infection prevalence or higher PRR. The structure of the linear mixed-effects model used is shown in Appendix A1.

All the statistical analyses were carried out using STATA version 15.1 (STATA Corporation, College Station, TX), and all graphs were developed using the *ggplot* package implemented in *R*.^30^

## RESULTS

### Summary of the program impact after 5 years of implementation.

**Figure 1.**
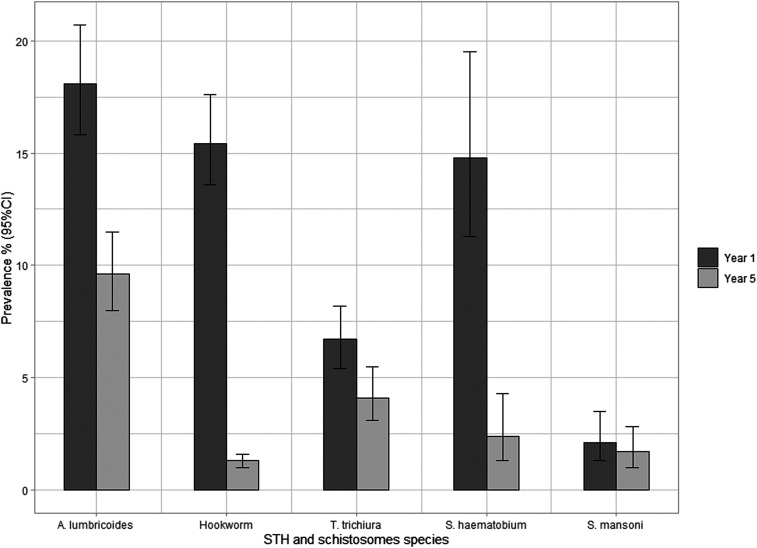
Comparison of year 1 and year 5 infection prevalence for soil-transmitted helminths (STH) and schistosome species among the 199 schools included in the analysis. The prevalence together with the associated 95% CI was estimated using binomial regression model while accounting for school clusters.

Detailed analysis of the NSBD program impact after 5 years of implementation is presented elsewhere by Mwandawiro et al*.*^8^ However, in summary, among the 199 schools included in this current analysis and surveyed during year 1 (2012) and year 5 (2017), combined STH prevalence was 32.3% (*A. lumbricoide*s 18.1%, hookworm 15.4%, and *T. trichiura* 6.7%) during year 1, and was 13.5% (*A. lumbricoides* 9.6%, hookworm 1.3%, and *T. trichiura* 4.1%) after 5 years. Accordingly, the schistosomiasis prevalence was 14.8% and 2.1% during year 1, and 2.4% and 1.7% during year 5 for *S. haematobium* and *S. mansoni*, respectively (Figure 1). The associated 5-year PRR was 58.2% (*P* < 0.001), 46.8% (*P* < 0.001), 91.6% (*P* < 0.001), and 38.4% (*P* < 0.001) for STH combined, *A. lumbricoides*, hookworm, and *T. trichiura*, respectively, and 84.0% (*P* < 0.001) and 19.3% (*P* = 0.062) for *S. haematobium* and *S. mansoni*, respectively.

On average, the whole country has received four rounds of treatment (range: 3–7 rounds) for albendazole and one round (range: 0–5 rounds) for praziquantel over the last 5 years. The average treatment rounds for the NSBD program areas (i.e., areas treated for STH/schistosomiasis only by the NSBD program) were also four rounds (range: 3–4 rounds) for albendazole and one round (range: 0–5 rounds) for praziquantel. Therefore, areas treated by both the LF and NSBD program using albendazole, which include counties in the coastal region, had achieved an average of seven rounds (range: 6–7 rounds) within the 5-year period. From these results, we see that either some schools analyzed had missed out on the regular treatment program or the treatment was not delivered in some program years. Therefore, in this current analysis, we present the results of the school-level associations between infection prevalence and treatment coverage and other factors related to WASH and environment.

### Univariable analysis between infection prevalence and water, sanitation, and hygiene; treatment coverage; and environmental influences.

Univariable analysis was conducted on each of the two outcomes, infection prevalence and PRR, for each STH and schistosome species. Supplemental Tables S2 and S3 provide the details of univariable analysis results for each STH and schistosome species, respectively.

In the univariable analysis for *A. lumbricoides*, of the 25 variables considered, seven were significantly associated with reduced school-level *A. lumbricoides* prevalence and two were significantly associated with increased school-level prevalence. Reduced *A. lumbricoides* prevalence in year 5 was significantly associated with low (< 1%) baseline prevalence, a high (seven rounds) number of rounds of treatment, and areas treated by both LF and NSBD programs. In terms of environmental features, reduced *A. lumbricoides* prevalence was significantly associated with 25°C of LST, 20–70 mm/hour of precipitation (showers of rainfall), and low elevation of the school (< 500 m; near sea level altitude). Increased *A. lumbricoides* prevalence in year 5 was significantly associated with high (≥ 1,000 pupils) school population, and a high AI (hyper-humidity). On the other hand, lower PRR was significantly associated with low (< 1%) baseline prevalence, whereas higher PRR was significantly associated with clean latrines at school and low elevation of the school (< 500 m; near sea level altitude; Supplemental Table S2).

In the univariable analysis for hookworms, reduced prevalence in year 5 was significantly associated with the presence of a school feeding program. Increased prevalence was significantly associated with a high (60–90) pupils per latrine ratio, 20–25°C LST, and the school being on a moderately steep slope. Looking at PRR as an outcome, lower PRR was significantly associated with a high (60–90) pupils per latrine ratio, whereas higher PRR was significantly associated with four rounds of treatment (Supplemental Table S2).

Univariable analysis for *T. trichiura* showed that reduced risk of prevalence in year 5 was significantly associated with low (< 1%) baseline prevalence, high (50–75%) reported coverage of household improved water source, and school population of 200–499 pupils. Increased risk of *T. trichiura* prevalence was not significantly associated with any of the evaluated covariates, but was mildly nonsignificantly associated with a high (60–90 pupils) pupils per latrine ratio and community population density of 5–10 people per 100 m^2^. In terms of PRR, lower PRR was significantly associated with high (50–75%) reported coverage of a household handwashing facility equipped with water and soap. Higher PRR was significantly associated with areas treated by both LF and NSBD programs, and areas with more than 25°C of LST (Supplemental Table S2).

Results for univariable analysis for *S. mansoni* indicated that reduced prevalence in year 5 was significantly associated with baseline prevalence, with a gradient effect observed from low (< 1%) baseline prevalence, 1–10% baseline prevalence, and 10–50% baseline prevalence. Increased prevalence was significantly associated with moderately low (25–50%) reported coverage of a household improved water source, LST of 20–25°C, clay soil type, community population density of 5–10 people per 100 m^2^, 4+ treatment rounds, 75–85% treatment coverage, the presence of a school feeding program, and the presence of a school sanitation program. From the results, lower PRR was significantly associated with one treatment round, and 50–75% reported coverage of a household handwashing facility equipped with water and soap. Higher PRR was significantly associated with a low pupils per latrine ratio (< 30 pupils) (Supplemental Table S3).

Univariable analyses for *S. haematobium* indicated that reduced prevalence in year 5 was significantly associated with clay soil type, whereas increased prevalence was significantly associated with a low number (two) of treatment rounds, high AI (hyper-humid), and availability of a school handwashing facility equipped with water and soap. From the results, lower PRR was significantly associated with baseline prevalence of 1–10%, whereas higher PRR was significantly associated with the availability of school handwashing facility equipped with water and soap, and areas receiving 20–70 mm/hour of precipitation (Supplemental Table S3).

### Multivariable analysis between infection prevalence and water, sanitation, and hygiene; treatment coverage; and environmental influences.

Before conducting multivariable analysis, pairwise correlation analysis was performed on all the continuous covariates selected for inclusion in the multivariable models to determine if strong (*r* ≥ 0.70 or *r* ≤ −0.70) collinearity existed among the variables. From the results of this pairwise correlation analysis (Supplemental Table S1), strong collinearity existed between AI and proportion of households with improved water source (*r* = −0.721), elevation and proportion of households with improved water source (*r* = −0.704), and elevation and LST (*r* = −0.988). Therefore, effort was made to ensure that no two collinear covariates were included in a single multivariable model.

Results from the multivariable model for *A. lumbricoides* indicated that reduced risk for school-level infection prevalence in year 5 was significantly associated with baseline prevalence, with a gradient effect observed from low (< 1%) baseline prevalence (coefficient [*C*] = −28.16, *P* < 0.001), 1–20% baseline prevalence (*C* = −25.80, *P* < 0.001), and 20–50% baseline prevalence (*C* = −13.55, *P* < 0.001), compared with > 50% baseline prevalence. Also gradient effect with treatment rounds was observed, from four rounds (*C* = −12.64, *P* < 0.001), six rounds (*C* = −24.60, *P* < 0.001), and seven rounds (*C* = −27.29, *P* < 0.001), compared with three rounds. Furthermore, reduced prevalence was associated with high (50–75%) self-reported coverage of a household handwashing facility equipped with water and soap compared with < 25% reported coverage (*C* = −7.06, *P* = 0.012), 20–25°C of LST compared with < 20°C (*C* = −4.99, *P* = 0.015), and community population density of 5–10 people per 100 m^2^ compared with < 5 people per 100 m^2^ (*C* = −4.16, *P* = 0.049). Increased prevalence was associated with school population of ≥ 1,000 pupils compared with < 200 pupils (*C* = 15.84, *P* < 0.001). In terms of PRR, lower PRR was associated with low (< 1%) baseline prevalence compared with > 50% baseline prevalence (*C* = −210.04, *P* = 0.001), whereas higher PRR was associated with low elevation of the school (< 500 m; near sea level altitude) compared with moderate altitude (2000–3000 m) (*C* = 254.61, *P* < 0.001) (Table 2).

**Table 2:** Multivariable analysis of factors associated with the school-level *A. lumbricoides* PRR among 199 schools surveyed during year 5 survey in Kenya

Covariate	*A. lumbricoides* (coefficient (95% CI), *P*-value)*	
	Prevalence	PRR
Baseline prevalence, %		
< 1	**−28.16 (−35.11;−21.20), *P* < 0.001**	**−210.04 (−329.29;−90.80), *P* = 0.001**
1–20	**−25.80 (−32.00;−19.60), *P* < 0.001**	−26.49 (**−**117.93;64.96), *P* = 0.570
20–50	**−13.55 (−19.19;−7.91), *P* < 0.001**	−25.59 (**−**66.59;117.77), *P* = 0.586
> 50	Reference	
Treatment covariates		
Treatment rounds with ABZ		
Three	Reference	
Four	**−12.64 (−18.48;−6.80), *P* < 0.001**	
Six	**−24.60 (−35.04;−14.17), *P* < 0.001**	
Seven	**−27.29 (−36.23;−18.36), *P* < 0.001**	
Household WASH covariates		
Proportion of children reporting handwashing facility with soap and water at home, %		
< 25	Reference	
25–50	−4.04 (**−**9.52;1.43), *P* = 0.147	
50–75	**−7.06 (−12.57;−1.56), *P* = 0.012**	
> 75	Insufficient obs	
School WASH covariates		
School population (pupils)		
< 200	Reference	
200–499	**8.64 (1.96;15.31), *P* = 0.011**	
500–999	6.30 (**−**0.39;12.98), *P* = 0.065	
≥ 1,000	**15.84 (7.22;24.47), *P* < 0.001**	
Environmental covariates		
Land surface temperature (°C)		
< 20	Reference	
20–25	**−4.99 (−9.02;−0.96), *P* = 0.015**	
> 25	Insufficient obs	
Elevation (in meters)		
< 500 (near sea level)		**254.61 (155.54;353.68), *P* < 0.001**
500–2000 (low altitude)		**143.75 (71.19;216.32), *P* < 0.001**
2000–3,000 (moderate altitude)		Reference
Population density (per 100m^2^)		
< 5	Reference	
5–10	**−4.16 (−8.30;−0.02), *P* = 0.049**	
≥ 10	−1.15 (**−**6.19;3.90), *P* = 0.656	

*A. lumbricoides* = *Ascaris lumbricoides*; PRR = prevalence relative reduction; WASH = water, sanitation, and hygiene.

* Regression coefficients of association together with their 95% CIs were determined using multivariable mixed-effects linear regression models with a random intercept at county and subcounty levels. Statistical significance of the coefficients was determined by the absence of zero overlapping in the 95% CIs (values in bold). In all the models, negative values of the coefficients indicated a decrease in the rate of school-level infection or low values of PRR, whereas positive values of the coefficients indicated an increase in the rate of school-level infection or high values of PRR.

Multivariable analysis of hookworms indicated that reduced risk for school-level infection prevalence in year 5 was significantly associated with low (< 1%) baseline prevalence compared with > 50% baseline prevalence (*C* = −3.10, *P* < 0.001) and a school feeding program (*C* = −1.09, *P* = 0.005), whereas increased risk was significantly associated with a high (60–90) pupils per latrine ratio compared with a very high (> 90 pupils) ratio (*C* = 2.11, *P* < 0.001). In terms of PRR, lower school-level PRR was significantly associated with a high (60–90) pupils per latrine ratio compared with a very high (> 90 pupils) ratio (*C* = −50.78, *P* < 0.001), whereas higher school-level PRR was significantly associated with seven rounds of treatment compared with three rounds (*C* = 27.23, *P* = 0.042; Table 3).

**Table 3:** Multivariable analysis of factors associated with the school-level hookworm PRR among 199 schools surveyed during year 5 survey in Kenya

Covariate	Hookworm (Coefficient (95% CI), *P*-value)*	
	Prevalence	PRR
Baseline prevalence, %		
< 1	**−3.10 (−4.46;−1.75), *P* < 0.001**	
1–20	**−2.09 (−3.07;−1.11), *P* < 0.001**	
20–50	Insufficient obs	
> 50	Reference	
Treatment covariates		
Treatment rounds with ABZ		
Three		Reference
Four		20.16 (**−**0.86;47.08), *P* = 0.059
Six		26.76 (**−**6.88;68.10), *P* = 0.110
Seven		**27.23 (1.13;65.41), *P* = 0.042**
School WASH covariates		
Overall pupil per latrine ratio		
< 30	**1.47 (0.59;2.36), *P* = 0.001**	−10.53 (**−**30.29;8.89), *P* = 0.285
30–60	**1.28 (0.50;2.07), *P* = 0.001**	−2.07 (**−**21.64;14.65), *P* = 0.706
60–90	**2.11 (0.97;3.25), *P* < 0.001**	**−50.78 (−76.76;−22.85), *P* < 0.001**
> 90	Reference	
School feeding program	**−1.09 (−1.85;−0.33), *P* = 0.005**	

PRR = prevalence relative reduction.

* Regression coefficients of association together with their 95% CIs were determined using multivariable mixed-effects linear regression models with a random intercept at county and subcounty levels. Statistical significance of the coefficients was determined by the absence of zero overlapping in the 95% CIs (values in bold). In all the models, negative values of the coefficients indicated a decrease in the rate of school-level infection or low values of PRR, whereas positive values of the coefficients indicated an increase in the rate of school-level infection or high values of PRR.

Multivariable analysis of *T. trichiura* indicated that reduced risk for school-level infection prevalence in year 5 was significantly associated with low (< 1%) baseline prevalence compared with > 50% baseline prevalence (*C* = −47.25, *P* < 0.001), whereas increased risk was significantly associated with low (25–50%) self-reported coverage of a household improved water source compared with < 25% reported coverage (*C* = 9.26, *P* < 0.001) and a gradient effect on high (60–90) number of pupils per latrine ratio compared with > 90 ratio (*C* = 7.88, *P* = 0.001). In terms of PRR, high (50–75%) self-reported coverage of a household handwashing facility equipped with water and soap compared with < 25% reported coverage was the only significant factor associated with lower school-level PRR (*C* = −73.65, *P* = 0.030; Table 4).

**Table 4:** Multivariable analysis of factors associated with the school-level *T. trichiura* prevalence among 199 schools surveyed during year 5 survey in Kenya

Covariate	*T. trichiura* (coefficient (95% CI), *P*-value)* prevalence
Baseline prevalence, %	
< 1	**−47.25 (−57.68;−36.82), *P* < 0.001**
1–20	**−44.07 (−54.64;−33.50), *P* < 0.001**
20–50	**−28.15 (−38.67;−17.63), *P* < 0.001**
> 50	Reference
Household WASH covariates	
Proportion of children reporting an improved household water source at home, %	
< 25	Reference
25–50	**9.26 (5.52;13.01), *P* < 0.001**
50–75	−2.40 (**−**6.49;1.69), *P* = 0.250
> 75	−0.53 (**−**3.77;2.70), *P* = 0.746
School WASH covariates	
Overall pupil per latrine ratio	
< 30	**3.98 (0.74;7.22), *P* = 0.016**
30–60	**5.78 (2.92;8.65), *P* < 0.001**
60–90	**7.88 (3.41;12.34), *P* = 0.001**
> 90	Reference

*T. trichiura* = *Trichuris trichiura*; PRR = prevalence relative reduction; WASH = water, sanitation, and hygiene.

* Regression coefficients of association together with their 95% CIs were determined using multivariable mixed-effects linear regression models with a random intercept at county and subcounty levels. Statistical significance of the coefficients was determined by the absence of zero overlapping in the 95% CIs (values in bold). In all the models, negative values of the coefficients indicated a decrease in the rate of school-level infection or low values of PRR, whereas positive values of the coefficients indicated an increase in the rate of school-level infection or high values of PRR.

Reduced risk of school-level *S. mansoni* prevalence in year 5 was significantly associated with low (< 1%) baseline prevalence compared with > 50% baseline prevalence (*C* = −48.95, *P* < 0.001), three treatment rounds compared with zero rounds (*C* = −5.91, *P* = 0.003), and high (> 75%) self-reported coverage of a household improved water source compared with < 25% reported coverage (*C* = −1.70, *P* = 0.015). In terms of PRR, lower school-level PRR was significantly associated with a low (one) number of treatment rounds compared with zero rounds (*C* = −380.59, *P* = 0.001; Table 5).

**Table 5:** Multivariable analysis of factors associated with the school-level *S. mansoni* PRR among 199 schools surveyed during year 5 survey in Kenya

Covariate	*S. mansoni* (Coefficient (95% CI), *P*-value)*	
	Prevalence	PRR
Baseline prevalence, %		
< 1	**−48.95 (−54.79;−43.12), *P* < 0.001**	
1–10	**−43.58 (−49.94;−37.23), *P* < 0.001**	
10–50	**−37.14 (−43.03;31.24), *P* < 0.001**	
> 50	Reference	
Treatment covariates		
Treatment rounds with PZQ		
0	Reference	
One	2.49 (**−**4.77;9.77), *P* = 0.500	**−380.59 (−610.36;−150.81), *P* = 0.001**
Two	**−6.73 (−12.39;−1.06), *P* = 0.020**	−143.78 (**−**387.49;99.93), *P* = 0.248
Three	**−5.91 (−9.78;−2.04), *P* = 0.003**	**−165.68 (−301.08;−30.29), *P* = 0.016**
≥ Four	Insufficient obs	**−298.19 (−446.50;−149.87), *P* < 0.001**
Household WASH covariates		
Proportion of children reporting an improved household water source at home, %		
< 25	Reference	
25–50	1.47 (**−**0.33;3.28), *P* = 0.110	
50–75	−0.82 (**−**2.82;1.18), *P* = 0.424	
> 75	**−1.70 (−3.40;0.01), *P* = 0.015**	
School WASH covariates		
Overall pupil per latrine ratio		
< 30		104.78 (**−**22.23;231.79), *P* = 0.106
30–60		−128.74 (**−**277.05;19.58), *P* = 0.089
60–90		65.99 (**−**138.45;270.44), *P* = 0.527
> 90		Reference

*S. mansoni* = *Schistosoma mansoni*; PRR = prevalence relative reduction; WASH = water, sanitation, and hygiene.

* Regression coefficients of association together with their 95% CIs were determined using multivariable mixed-effects linear regression models with a random intercept at county and subcounty levels. Statistical significance of the coefficients was determined by the absence of zero overlapping in the 95% CIs (values in bold). In all the models, negative values of the coefficients indicated a decrease in the rate of school-level infection or low values of PRR, whereas positive values of the coefficients indicated an increase in the rate of school-level infection or high values of PRR.

Reduced risk of school-level *S. haematobium* prevalence in year 5 was significantly associated with high AI (hyper-humid) compared with semiaridity (*C* = −3.32, *P* = 0.025), whereas increased risk was significantly associated with a low (two) number of treatment rounds compared with zero rounds (*C* = 2.58, *P* = 0.029). In terms of PRR, higher school-level PRR was significantly associated with 1–10% baseline prevalence compared with > 50% baseline prevalence (*C* = 10.25, *P* = 0.001) and low precipitation of between 20 and 70 mm/hour (showers of rainfall) compared with 70–280 mm/hour (thunderstorm rainfall) (*C* = 39.43, *P* < 0.001; Table 6).

**Table 6:** Multivariable analysis of factors associated with the school-level *S. haematobium* PRR among 199 schools surveyed during year 5 survey in Kenya

Covariate	*S. haematobium* (Coefficient (95% CI), *P*-value)*	
	Prevalence	PRR
Baseline prevalence, %		
< 1		Insufficient obs
1–10		**10.25 (4.27;16.23), *P* = 0.001**
10–50		Insufficient obs
> 50		Reference
Treatment covariates		
Treatment rounds with PZQ		
Zero	Reference	
One	−0.02 (**−**2.09;2.05), *P* = 0.988	
Two	**2.58 (0.27;4.89), *P* = 0.029**	
Three	1.42 (**−**0.19;3.02), *P* = 0.083	
≥ Four	Insufficient obs	
Environmental covariates		
Aridity index†		
< 0.50 (semiarid)	Reference	
0.50–0.65 (dry sub-humid)	−1.02 (**−**2.71;0.66), *P* = 0.235	
0.65–0.75 (humid)	−1.09 (**−**2.85;0.67), *P* = 0.226	
> 0.75 (hyper-humid)	**−3.32 (−6.23;−0.41), *P* = 0.025**	
Precipitation (in mm/hour)		
20–70 (showers rainfall)		**39.43 (32.28;46.58), *P* < 0.001**
70–280 (thunderstorm rainfall)		Reference

*S. haematobium* = *Schistosoma haematobium*; PRR = prevalence relative reduction; WASH = water, sanitation, and hygiene.

* Regression coefficients of association together with their 95% CIs were determined using multivariable mixed-effects linear regression models with a random intercept at county and subcounty levels. Statistical significance of the coefficients was determined by the absence of zero overlapping in the 95% CIs (values in bold). In all the models, negative values of the coefficients indicated a decrease in the rate of school-level infection or low values of PRR, whereas positive values of the coefficients indicated an increase in the rate of school-level infection or high values of PRR.

† Classification of the values of aridity index was adapted from the UN Environment Programme (UNEP) (https://www.unenvironment.org/).

## DISCUSSION

Most research efforts within Kenya have previously focused solely on individual-level factors influencing the epidemiology of STH and schistosomiasis,^19,31–34^ with little focus to school location factors that may influence the infection variation over time within small areas due to environmental variables such as rainfall, land cover, soil type, aridity, elevation, and LST, among other factors. Here, we have evaluated, for the first time within a national program, school location factors that may influence transmission dynamics in Kenya. This information is helpful in giving a detailed context of the key factors driving the heterogeneity in infection prevalence in some areas.

We examined these associations using school-level data for two main outcomes, infection prevalence and 5-year PRR, across several domains: 1) individual worm species; *A. lumbricoides*, hookworm, *T. trichiura*, *S. mansoni*, and *S. haematobium*. 2) WASH exposures at school and community where the pupils learn or live, 3) treatment coverage and MDA rounds, and 4) environmental exposures around the school location. This is helpful, given that the different helminths have different pathways of exposure. Care should be taken in interpreting the regression coefficients for the two outcomes: prevalence and PRR; we reiterate that throughout the models, negative values of the coefficients indicated a decrease in the school-level infection prevalence or lower PRR, whereas positive values of the coefficients indicated an increase in the school-level infection prevalence or higher PRR. It is easy to see that low values of PRR means that the year 5 infection prevalence did not reduce much from year 1, especially when the baseline prevalence was already low. Hence, low values of PRR can conversely imply that there is still substantially high infection prevalence at year 5. High values of PRR implied a greater reduction of prevalence from year 1 to year 5; this is true especially if the factors favoring prevalence reduction, such as a high number treatment rounds, were present. Hence, high values of PRR can indicate that there is reduced infection prevalence at year 5.

In Kenya, the spatial distribution of these infections in different regions and counties is well established,^19,35–38^ and is believed to be influenced by diverse factors such as living and socioeconomic conditions (e.g., type of building structure and access to WASH facilities), environmental and climatic conditions (e.g., soil type, elevation, and rainfall) that influence individual behaviors, and infection transmission dynamics. Herein, we objectively quantified some of these factors’ exposure to school-level infection at a national scale.

The high number of treatment rounds was significantly associated with reduced infection prevalence, and consequently high PRR values, for *A. lumbricoides* and hookworm but not for *T. trichiura*. It is important to note that helminthic treatment in most parts of the country is usually performed annually using single-dose oral albendazole (for STH and LF) and praziquantel (for schistosomiasis). Therefore, this finding is consistent with several other studies that have shown tremendous efficacy of single-dose oral anthelminthic drugs such as mebendazole and albendazole toward *A. lumbricoides* and hookworm but not *T. trichiura*,^39–43^ which perhaps require high treatment coverage coupled with prolonged 3-day dosing regimen of albendazole,^44^ or drug combination of albendazole or mebendazole coadministered with ivermectin.^45^ As such, the high number of treatment rounds, per se, does not appear to be sufficient to reduce the prevalence of *T. trichiura*. However, the number of treatment rounds delivered showed mixed impacts when it comes to schistosomiasis. Although more than three rounds suggested reduced *S. mansoni* infection, it did not reveal the same for *S. haematobium*, which increased with the low number of treatment rounds. This could be explained by the occasional irregular and inconsistent delivery of praziquantel drug by the Kenyan national NTD control program.^8^

Categorization of the country according to different treatment programs, that is, areas treated by NSBD only, and those co-treated by other partners, unmasked the usually assumed NSBD program-wide impact. As such, areas treated by both NSBD and other partner programs showed significant reduction of prevalence, especially for *A. lumbricoides*. In addition, it showed significantly high PRR values for *T. trichiura*. The cumulative effect of multiple related program interventions on the same areas covered by the NSBD program was the increased treatment coverage and rounds. Again, the effect could have not been more pronounced on other STH species because the single-dose albendazole drug used by most of these partner programs is usually less efficacious against *T. trichiura*^40^; in addition, hookworm prevalence at the year 5 survey could have been too low to show any significant association. Consequently, these additional programs’ participation did not seem to add any treatment benefit to schistosomiasis; this was mainly true because none of them delivered praziquantel drug.

Child-reported community-level factors such as high coverage of household handwashing facilities equipped with water and soap and improved household water source were vital in providing community protection against *A. lumbricoides* and equally *T. trichiura*, respectively. The importance of these WASH conditions in decelerating the infection burden, especially for STH, cannot be overemphasized as several studies have indicated their role as complementary interventions necessary, alongside chemotherapy, in accelerating the attainment of NTD elimination.^46–49^ From this result, we can infer that these two WASH conditions are interdependent and effectively provide stronger protection when they are accessible together. For instance, effective handwashing behavior may depend on community access to an improved water source that reliably supply water.^47^ We also noted that high-community population density increased *S. mansoni* prevalence. This finding agrees with a previous study carried out on the shores and islands of Lake Victoria, Kenya, that significantly associated it with *S. mansoni* infection risk.^38^ Theoretical modeling studies had previously determined that the basic reproduction number $\left(R_{o}\right)$ of schistosomiasis linearly increased with human density.^50–52^ The influence of the population density on the *S. mansoni* infection risk can be explained by the numerical dynamics of the transmission and by the fact that densely populated areas, which often have poorly managed sewerage system, mean greater availability of human hosts and, if coupled by poor hygiene behaviors (e.g., human defecation or urination directly into water bodies), promote access of *Schistosoma* miracidiae to snails.^53,54^ We therefore strongly recommend safe management of wastewater in densely populated areas/urban settings as well as proper development of the urban physical environment.

Interviewer-observed school conditions such as high population of pupils in a school and high number pupils per latrine increased the prevalence for all the STH species. This finding is supported by other studies that have reported possible impacts of high population in schools and a high number of pupils per latrine as occasioned by increased dirtiness in the latrines due to overwhelming demand on the limitedly available latrines, which can lead to increased fecal exposure and contamination.^55,56^ Other studies have warned that simply meeting the pupils to latrine ratio in the absence of reduction on school population and disease-exposure pathways may be insufficient to improve health.^57,58^ In addition, we observed that availability of the school feeding program was associated with significantly reduced hookworm prevalence. Usually, school feeding programs have been shown to improve nutritional status of the schoolchildren and positively impact their health and educational outcomes.^59^ The positive impact of these programs on parasitic infection control can be linked to the epidemiology of the infections and the fact that they can cause serious nutritional deficiency and contribute to anemia,^60^ a key characteristic of hookworm infection. As such, proper nutrition can be key in suppressing devastating effects of helminths and schistosomiasis.

Our assessment of the above community and school WASH conditions helped to gauge the country’s progress toward reducing inequalities in WASH services at both household and school levels among the disadvantaged segment of the population (schoolchildren). Our reporting focused on the following long-standing WASH-sector objectives, which are also reflected in the global sustainable development goal (SDG) targets and indicators related to WASH: 1) improving access to safe and affordable drinking water, and 2) improving access to adequate sanitation and hygiene and ending open defecation.^61^ It is important to note that achieving these universal SDG targets requires faster progress among these disadvantaged groups.^62^

The inclusion of the environmental factors in the models quantified insightful results toward infection transmission dynamics for different species in Kenya. In short, environmental exposure due to school location appeared to be a stronger determinant of infection than individual characteristics.^38,63^ From our models, we were able to show that reduced *A. lumbricoides* infection was associated with high LST of above 25°C, moderately low amount of precipitation (of 20–70 mm/hour), and low elevation (near sea level). High AI was associated with increased *A. lumbricoides* but reduced *S. haematobium*. Moderate (20–25°C) LST and moderately steep slope were associated with increased hookworm prevalence, whereas increased *S. mansoni* prevalence was associated with high LST and clay soil type. The environmental associations presented here are consistent with the known biological determinants of helminth transmission.^64–66^ Actually, past experimental studies have categorically shown that the development of free-living infectious stages of *A. lumbricoides* and *T. trichiura* die off at 38°C and hookworm at 40°C.^35,67–69^ This is further supported by a large-scale geospatial study that illustrated the relationship between STH prevalence across Kenya and maximum LST,^35^ from that study and other related studies; areas masked as unsuitable for STH transmission had LST > 40°C.^68^ Furthermore, rigorous geostatistical variable selection methods have in the past identified specific environmental and ecological determinants that govern the helminth geographical distribution and lifecycles in a complex way in various regions.^69,70^ From these studies, the distribution of *A. lumbricoides* was positively associated with high precipitation above 400 mm and hyper-humidity.^70^ This is true because high humidity has been associated with the faster development of parasite eggs in the free environment, whereas low humidity was seen as unfavorable for embryonation of *A. lumbricoides*.^71^ Past studies have indicated that too much precipitation (rain) is unfavorable to the infectious materials in the environment because the rain may carry them away in the runoff.^72,73^ The observation that moderately steep slope and soil type may enhance infection risk is supported by other environmental studies that have suggested that migration of the infectious materials can be faster on a sloppy landscape, where they can move as deep as 15 cm and as far as 40 cm from the center of fecal pat.^72^

To conclude, we believe that this kind of robust assessment of the associated factors influencing the transmission dynamics of STH and schistosomiasis, although not casual in nature, offers a general indication of the school and community environments where children learn and live in. This assessment prides in the use of large-scale deworming program data like the Kenyan NSBD, and can be critical in influencing evidence-based policy decisions to the control programs, nationally and globally, as the world rallies toward the elimination goal.

### Study limitations.

We acknowledge some limitations of our study. First, a detailed WASH questionnaire was only administered during year 6 survey, approximately 12 months after the year 5 survey. This introduced a time gap between year 5 prevalence data and some of the WASH data. The use of these year 6 WASH data further introduced some missingness on some of the WASH variables because during year 6, only 100 schools instead of 199 were surveyed because of change on the M&E design. Second, although we recorded no evidence for bias caused by self-reported WASH data, the possibility of existence of such bias is a further limitation.

## CONCLUSION

Our findings show evidence that school-level prevalence, especially for STH infections, was strongly influenced by environmental conditions such as LST, precipitation, elevation, and AI. The presence of schistosomes, especially *S. mansoni*, was influenced by the type of soil. In addition, other factors such as the low number of treatment rounds, community coverage of handwashing facilities and improved water source, high population density both at community and school, and a high number of pupils to latrine ratio were significantly associated with increased infections. Hence, for sustainable control and elimination of these infections, going forward, refining and designing of programmatic interventions need to address the inclusion of the aforementioned factors.

## Supplemental tables and figures

Supplemental materials

## Supplemental Appendix

A1: The association coefficients between school-level infection prevalence or RR (PRR) were separately estimated using a linear mixed-effects regression model of the following mathematical structure:

$$y_{i}=X_{i}b+Z_{i}v_{i}+\varepsilon_{i};for\;i=1,2,\ldots ,199,$$

where $y_{i}$ is the ith subject response vector (i.e., school level year 5 prevalence or PRR), $X_{i}$ is the fixed-effects design matrix (i.e., predictor variables related to treatment, WASH, and environmental variables that were included in the model), b is the fixed-effects vectors (i.e., coefficients related to the fixed effects), $Z_{i}$ is the random-effects design matrix (i.e., counties and subcounties included as random intercepts), $v_{i}$ is the random-effects vector (i.e., coefficients related to the random effects), and $\varepsilon_{i}$ is the error term vector.
