# Supplementary material for: Statistical Regression Model of Water, Sanitation, and Hygiene; Treatment Coverage; and Environmental Influences on School-Level Soil-Transmitted Helminths and Schistosome Prevalence in Kenya: Secondary Analysis of the National Deworming Program Data
Source: Am J Trop Med Hyg. 2021 Apr 12;104(6):2251–63. doi: 10.4269/ajtmh.20-1189 (PMC8176504; doi:10.4269/ajtmh.20-1189)
Supplement: Supplementary file 1 [file tpmd201189.SD1.docx]

Appendix

A1: The association coefficients between school-level infection prevalence orRR(PRR) were separately estimated using a linear mixed-effects regression model of the following mathematical structure:

$y_{i}=X_{i}b+Z_{i}v_{i}+\varepsilon_{i};for i=1,2,\ldots,199$,

where$y_{i}$ is the $ith$ subject response vector (i.e., school level year 5 prevalence or PRR), $X_{i}$ is the fixed-effects design matrix (i.e., predictor variables related to treatment, WASH, and environmental variables that were included in the model), $b$ is the fixed-effects vectors (i.e., coefficients related to the fixed effects), $Z_{i}$ is the random-effects design matrix (i.e., counties and subcounties included as random intercepts), $v_{i}$ is the random-effects vector (i.e., coefficients related to the random effects), and $\varepsilon_{i}$ is the error term vector.
